# The Community As the Patient in Malaria-Endemic Areas: Preempting Drug Resistance with Multiple First-Line Therapies

**DOI:** 10.1371/journal.pmed.1001984

**Published:** 2016-03-29

**Authors:** Maciej F. Boni, Nicholas J. White, J. Kevin Baird

**Affiliations:** 1 Oxford University Clinical Research Unit, Wellcome Trust Major Overseas Programme, Ho Chi Minh City, Vietnam; 2 Centre for Tropical Medicine and Global Health, Nuffield Department of Medicine, University of Oxford, Oxford, United Kingdom; 3 Mahidol-Oxford Research Unit, Faculty of Tropical Medicine, Mahidol University, Bangkok, Thailand; 4 Eijkman-Oxford Clinical Research Unit, Jakarta, Indonesia

## Abstract

Maciej F. Boni and colleagues propose deploying multiple first-line combination therapies against malaria within a community to delay drug-resistance evolution.

**Summary Points**

- Combination therapy is an effective way to delay or prevent drug-resistance evolution in malaria, but we do not take full advantage of its potential.
- Deploying multiple first-line combination therapies allows us to challenge parasite populations with many different types of drugs, and thus delay and slow down drug-resistance evolution more than with a single combination therapy.
- We must take a preemptive, not reactive, policy approach to drug-resistance management in malaria.

## Combination Therapies

Among the chemotherapeutic tools used over the years in malaria control and elimination programs, combination therapies have been one of the most useful. Combining two drugs with differing mechanisms of action and, preferably, complementary pharmacokinetic properties both synergizes killing activity and protects against the emergence of resistance [1]. Excellent therapeutic efficacy has been demonstrated in many clinical trials, but the drug-resistance benefit—protecting against future resistance evolution—has not been formally assessed in the real world. Clinical trials aimed at such an endpoint would be unmanageably large in size and long in duration. Instead, we rightfully trust that a parasite population within any given patient will not have the numbers to overcome the odds against simultaneously developing the two unrelated genetic mechanisms needed to survive the killing mechanisms of both drugs in the combination. Eventually, of course, entire parasite populations across millions of human hosts do have those numbers and, under sufficient pressure and time, that double mutant will appear and proliferate. Nonetheless, by delaying this event, combination therapy increases the useful therapeutic life of individual drugs and treatments in clinical practice.

Artemisinin combination therapies (ACTs) have been increasingly used since 2005, when they were recommended as first-line treatments for falciparum malaria by the World Health Organization (WHO). The Global Fund and other donors then began supporting their adoption in place of the inexpensive and ineffective therapies used at that time. Just two years later, in 2007, evidence of partial resistance to artemisinins appeared at the Thai-Cambodian border [2], manifesting as a prolonged parasite clearance time in patients treated with a standard three-day course of ACT. Like other combination therapies, ACTs could not and did not stave off drug resistance forever. Why artemisinin resistance emerged then and there was unclear. One factor may have been the decades of unregulated use of artemisinin monotherapies before the appearance of ACTs. We will never know how long ACTs would have remained effective if artemisinin monotherapy use had been avoided altogether. A second contributory factor may have been resistance to the partner drugs coformulated in ACTs, e.g., the mefloquine component in artesunate-mefloquine (AS-MQ). AS-MQ was one of the first widely applied ACTs in the Thailand-Myanmar border areas, but in the 1990s mefloquine resistance, manifest as slower parasite clearance and a higher probability of recrudescence [3], spread throughout the region [4,5]. The end result was reduced mutual protection, and this lowered the probabilistic barrier to further resistance emergence.

Looking forward, the next potential advance in combination therapies is to raise the probabilistic barrier by combining three independent antimalarial drugs in a single treatment. An additional benefit of coformulated triple combinations is that the pharmacokinetic properties of two of the drugs will now be able to be matched, providing mutual protection to the non-artemisinin partner drugs, a crucial missing feature of current ACT formulations. A clinical trial testing triple combinations is currently recruiting in Southeast Asia [6]. In principle, if parasites are fully drug sensitive, a triple therapy presents the parasites with an almost insuperable problem: acquiring three independent genetic mechanisms simultaneously. The downside, of course, is that we risk an increase in side effects and costs, but on balance the benefits of preventing long-term drug-resistance evolution may well outweigh these risks. We have undervalued the benefits of combination therapy partly because they are difficult to see. The damage done by increased toxicity is immediate, as is the higher cost incurred by the patient, but the drug-resistance benefit of preserving these therapies is realized in the future. Maximizing these benefits takes active management. Strategies for the smart deployment of combination therapies have not been considered yet. Currently, all patients receive the same combination therapy, and this means that other combination therapies—if available—are held back.

A key element of malaria chemotherapy included in national malaria control program (NMCP) guidelines is the notion of “first-line” treatment. The first-line treatment is given to all patients unless contraindicated (e.g., allergy, pregnancy) or following therapeutic failure of the first-line treatment because of drug resistance or other causes. The WHO recommends NMCPs consider switching to a new first-line treatment if >10% of treatments fail. This is malaria treatment policy aimed at individual patients and the sum of their infecting parasites. This practice results in the parasite population as a whole in any given human community being confronted with a single therapeutic challenge—be it mono- or combined therapies—across patients and time. The primacy of first-line therapy ceases only when it inevitably fails.

## Multiple First-Line Therapies

**Fig 1:**
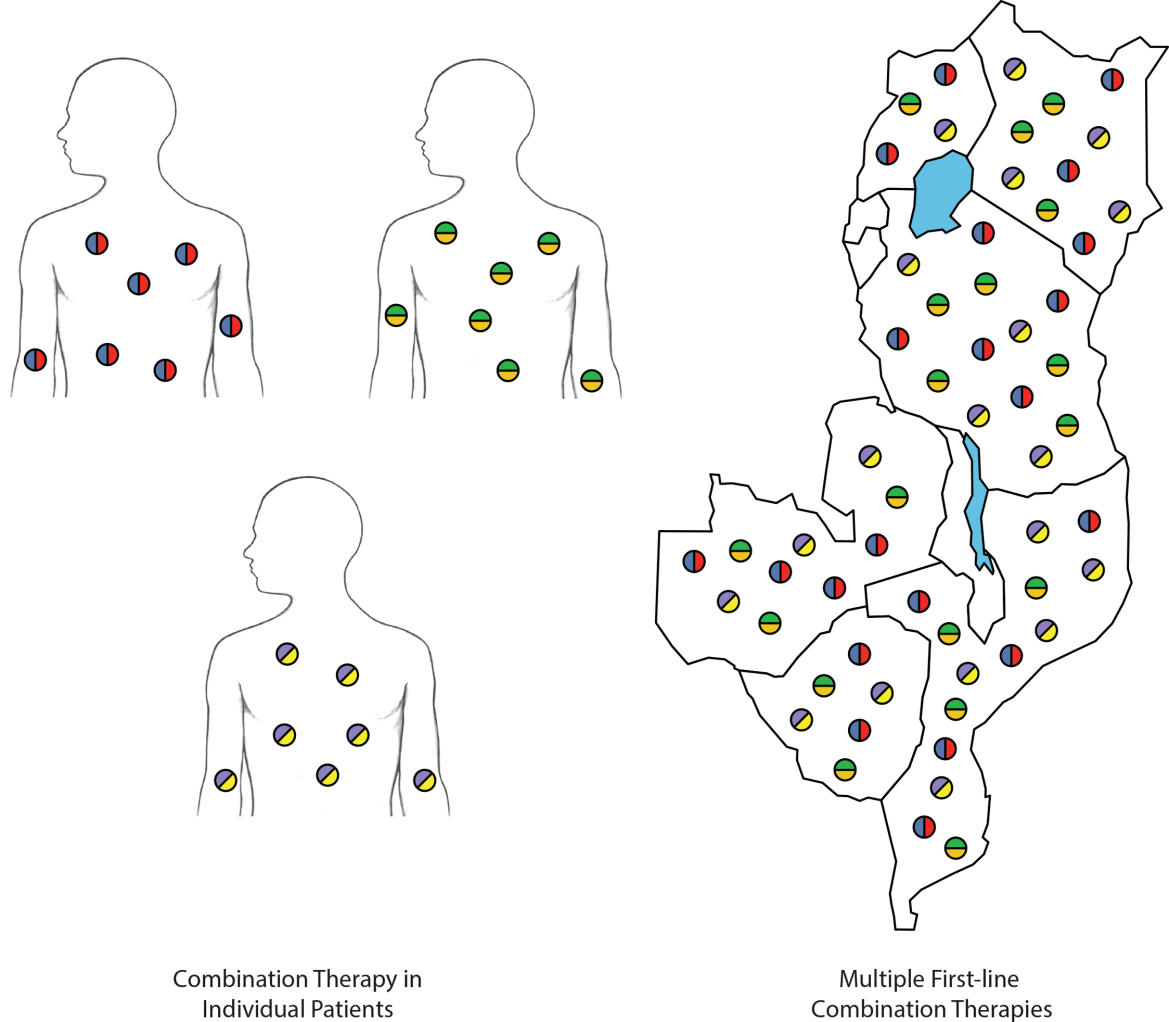
Methods of countering drug-resistance evolution. Individual patients treated with combination therapies (left). Deployment of multiple first-line combination therapies within and across countries (right). Each color represents a different antimalarial compound. In distributing multiple first-line combination therapies, the parasite population is challenged with more antimalarial compounds than when a single combination therapy is deployed.

Consider, instead, a malaria treatment strategy that targets the sum of parasites in a given human community, manifest in many human hosts over time. Rather than confronting that sum biomass with the challenge of acquiring two or perhaps three drug-resistance mechanisms, we could challenge it with the need to develop four or more phenotypes for survival across its human hosts, without needing to develop new antimalarial drugs. This can be achieved by distributing multiple chemotherapeutic challenges in communities of hosts—i.e., recommending the distribution of different first-line treatments for different patients (Fig 1). Provided that there is no cross-resistance, we can array three or four distinct treatments as equally weighted and applied “first-line” options. Over time, only parasites possessing resistance to all therapies will survive, but the risk of such an outcome is mitigated by its improbability.

The rationale for simultaneously distributing multiple equally efficacious therapies in a population comes from evolutionary theory. Darwinian adaptation is difficult in the face of multiple and shifting threats [7]. As an example, a parasite population in the bloodstream that has acquired lumefantrine resistance will not have any survival benefit if the next individual it infects is treated with dihydroartemisinin-piperaquine (DHA-PPQ). With three ACTs deployed equally in such a strategy, a lumefantrine-resistance mutation will have a one-in-three chance of experiencing a survivable environment in its next infection in a human host; cumulatively, these are not good odds. Under a scheme of simultaneous distribution of multiple first-line therapies (MFT), a parasite population in a community, over time, encounters multiple lethal threats to which it must quickly adapt or be annihilated. The time required to acquire and stabilize such a collection of genetic mechanisms may exceed the parasites’ ability to persist in communities applying this strategy.

As it is nearly impossible to run a field trial for an outcome that may or may not occur for a decade or longer, the current evidence in support of this theory is generated in silico using mathematical transmission models. These are rooted in original theoretical work demonstrating how multiple antimalarial drugs [8] or antibiotics [9] could be deployed to minimize the long-term detrimental effects of drug resistance. Under a policy of multiple first-line therapies, malaria transmission models indicate that drug resistance emergence will be delayed [10–13], and that subsequent drug resistance evolution will proceed more slowly [10,11] than under the status quo policy of using a single first-line therapy and replacing it when it fails. This evolutionary principle is supported by detailed modeling work focusing specifically on malaria epidemiology and distribution of ACTs [10]. The major concern for an MFT strategy is that multiple types of selection pressure will generate multi-drug–resistant parasites more quickly than if the therapies had been deployed one at a time [13], but this needs to be balanced against the process of genetic segregation (recombination breakdown), which breaks apart multi-drug–resistant genotypes as they pass through their diploid life stage in the mosquito. The population modeling analyses to date [10,11] do account for free recombination among resistance loci, but do not show evidence that deployment of MFT results in early emergence of multi-drug–resistant parasites.

## Implementation

Translating theory into policy and practice is challenging, to say the least. Initial challenges are likely to include planning for the right distribution method, managing its logistics, compensating for the higher costs of some therapies, and adjusting health-system delivery due to lower consumer preference for some drugs. However, NMCPs in many malaria-endemic settings have proven flexible and capable of managing such challenges. NMCPs already purchase and stockpile multiple types of drugs and insecticides, and insecticides are sometimes deployed in rotation to prevent resistance. In addition, NMCPs practice their own means of public health communication in implementing changes in health policy and practice. This depth of experience would surely be capitalized upon in easing the transition to MFT. Adopting an MFT approach may not be as difficult as thought, and additional benefits will become apparent as NMCPs gain experience managing its deployment. Under an MFT policy, new drugs or therapies can be incorporated into this system without disruption or major policy change.

A programmatic plan achieving an even distribution of multiple first-line therapies will need to be determined separately in each country or region. Possible strategies include a day-of-week prescription scheme (e.g., artemether-lumefantrine given for uncomplicated falciparum malaria on Mondays, DHA-PPQ on Tuesdays), a true randomization scheme (where possible), or a location-based scheme where different clinics or pharmacies are stocked with different antimalarial drugs. A pharmacy-based or clinic-based scheme has the particular advantage of allowing the management and distribution of multiple therapies to be done at a higher administrative level. A variety of distribution strategies have already been proposed, relying on the existence of different public health needs in antimalarial distribution (preventive, therapeutic) and multiple treatment formulations that are indicated by age or severity [14]. In many malaria-endemic countries, usage of antimalarials is already heterogeneous due to private sector drug purchasing [15,16]; this has the benefit of realizing population-level drug heterogeneity without an explicit policy, but the major disadvantage is that quality, antimalarial effectiveness, and uniformity of distribution cannot be easily controlled. A framework in which different therapies are allocated to private and public health networks would need to be managed actively to ensure the benefit of sufficient heterogeneity of antimalarial drug treatment.

## Population Chemotherapeutics: Preemptive Versus Reactive

There is a need for a more population-based approach to strategic drug resistance prevention in malaria. Using single first-line antimalarial therapies and devoting enormous resources to detecting the onset of resistance is a reactive rather than a preventive strategy. Most urgently, we should be focused on preserving artemisinin efficacy for as long as possible. In early 2015, the WHO’s Malaria Policy Advisory Committee reaffirmed the urgency and need for accelerated timelines in this endeavor [17]. In Southeast Asia—especially in regions where ACTs are now failing—preserving artemisinin amounts to eliminating *Plasmodium falciparum* as rapidly as possible and reducing the drug pressure on malaria parasites that allows for the continued selection and spread of resistance to all components of currently deployed ACTs; in Africa, preserving artemisinin would mean changing policy so that imported artemisinin-resistant genotypes face the least friendly environment possible. A policy of simultaneous arraying of multiple therapies creates the least favorable conditions for drug-resistance evolution. For artemisinin preservation, this may mean deploying a non-artemisinin–based therapy as a co-equal first-line therapy to be used alongside ACTs [10,14]. The major challenge, obviously, is finding such a therapy. In some parts of the world, this may be impossible due to the present high levels of drug-resistance to the most commonly available antimalarials; in other regions, a combination of currently available antimalarials would be a viable option. One way to select such a therapy would be based on opposing selection pressures that can be directly observed in clinical trials [18,19]. We may have a chance to slow down the spread of artemisinin resistance, but this would require acting very quickly at national and global levels of health policy.

Prevention and preemption should be the slogans of malaria drug-resistance planning. When or if new antimalarial compounds such as cipargamin (KAE609), the imidazolopiperazine KAF156, artefenomel (OZ439), and ferroquine pass through the necessary safety and efficacy trials and are adopted for wide distribution for treating uncomplicated malaria, they will be deployed as combinations, but they will still need to be introduced into the public health system in such a way that their presence does not immediately create substantial pressure for drug-resistant genotypes to evolve. The antimalarial effectiveness of these new compounds is a precious resource that must not be wasted [20]. These arguments suggest that the best way to preserve their efficacy is to introduce them alongside currently used therapies, rather than as a replacement for them. This will ensure that new drug-resistance mutations would emerge into an environment where they are immediately faced with an overwhelming variety of drug action from four or five different compounds. We can and we should manage the introduction of new antimalarials in such a way to extend their useful therapeutic life for as long as possible.

Achieving a varied drug distribution at the population level will allow us to stay one step ahead of the parasite. A paradigm of anticipation rather than reaction will allow us to keep prevalence levels low, drug resistance at bay, and policy focus on the long list of tasks necessary to move nations closer to their goals of malaria elimination. For many countries, malaria elimination may not be around the corner. With hundreds of millions of malaria cases per year globally, and challenging epidemiological, political, and economic situations in both African and Asian nations, global malaria policy should be looking thirty years into the future to ensure that our best efforts at elimination are not quickly undermined by strongly drug-resistant phenotypes. For artemisinin combination therapies, this danger is very explicit, with the possibility that the current partially resistant genotypes could evolve to a form of complete resistance, although this caution applies to any drug that is used too widely. Thirty years from now, we cannot be asking how to extricate ourselves from another epidemic of drug resistance. We must anticipate that event and lay the groundwork today to make the conditions for future drug resistance as unfavorable as possible. Adoption of MFT may achieve this. We need to view endemic malaria as the infection of a community in need of effective chemotherapeutic practice. The conventional clinical focus on the parasites infecting a particular patient is inadequate to the problem of long-term resistance management. The patient is the community, and the community parasite biomass is the infection in need of an effective and well-managed therapy.

## Abbreviations

ACT: Artemisinin combination therapy
AS-MQ: artesunate-mefloquine
DHA-PPQ: dihydroartemisinin-piperaquine
NMCP: national malaria control program
MFT: multiple first-line therapies
WHO: World Health Organization
